# Envenomation by the red-tailed coral snake (*Micrurus mipartitus*) in Colombia

**DOI:** 10.1186/s40409-017-0100-4

**Published:** 2017-02-14

**Authors:** Carlos A. Cañas, Fernando Castro-Herrera, Santiago Castaño-Valencia

**Affiliations:** 1 Department of Internal Medicine, Fundación Valle del Lili, Universidad Icesi, Cali, Colombia; 2 Department of Physiological Sciences, School of Health Sciences, Universidad del Valle, Cali, Colombia

**Keywords:** Red-tailed coral snake, *Micrurus mipartitus*, snake envenomation, Colombia

## Abstract

**Background:**

Although the red-tailed coral snake (*Micrurus mipartitus*) is widely distributed in Colombia and its venom is highly neurotoxic and life threatening, envenomation by this species is rare. Therefore, this report may shed some light on the clinical presentation of *M. mipartitus* bites.

**Case presentations:**

Herein, we describe two cases of patients bitten by red-tailed coral snakes, illustrating the clinical presentation of the victims, the outcomes and treatment provided.

**Conclusion:**

Envenomation caused by *M. mipartitus* provokes predicable neurotoxicity, and its treatment should be based on respiratory support and use of specific antivenom.

## Background

Envenomation caused by snake venoms remains a neglected public health problem in most tropical countries. A better understanding of the venoms and clinical aspects of snakebites facilitates the prevention and management of the victims [1, 2]. Colombia has a rich fauna of reptiles, particularly venomous snakes. The red-tailed coral snake *Micrurus mipartitus* is a member of the Elapidae family that is found in Panamá, Colombia, Ecuador and Venezuela. Despite its broad distribution in Colombia, envenomation is rare. About 0.4% of reported cases of envenomation by ophidians in the country are due to coral snakes [3]. Possible causes for this include low notification of cases, the evasive behavior of the snake and the small size of its mouth (with little proteroglyphous fangs).

**Fig. 1:**
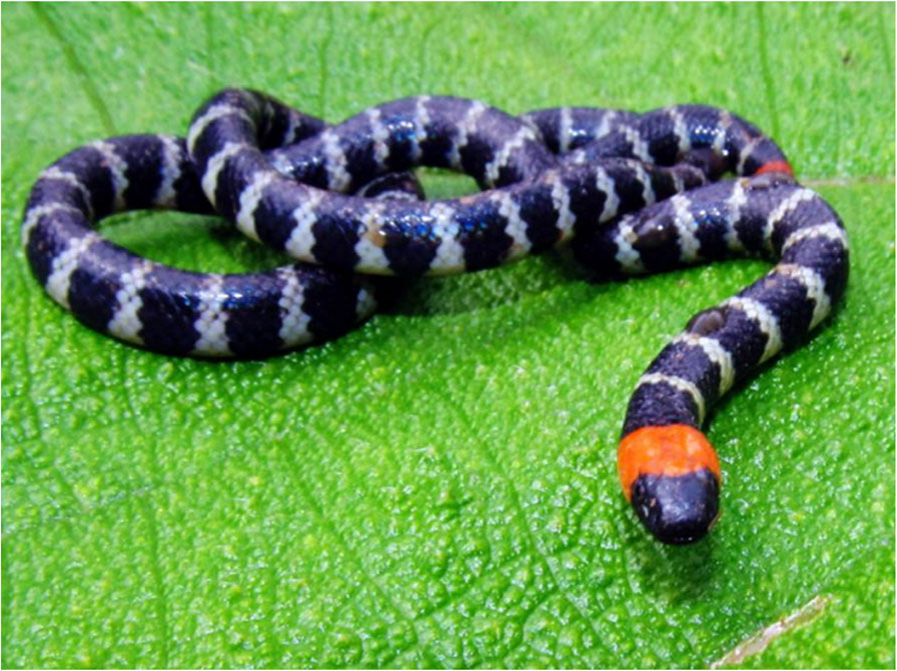
*Micrurus mipartitus* (red-tailed coral snake). Typical specimen with black body rings separated by white ones, except for the second head ring (red) and three or four of the tail rings

Accidents occur when the animal feels cornered or is harassed without opportunity to escape. *M. mipartitus* is common in agricultural areas of Colombia, especially coffee and sugarcane crops. It has also been seen on the Pacific Coast, the Western Oriental and Central Cordilleras, as well as in the Sierra Nevada de Santa Marta. They can be found in warm and cold climates, from 0 to 2,200 m above sea level. They can reach up 122 cm in length and have a cylindrical body, rounded small heads, very small eyes and short thick tails. Typical specimens have bright and smooth scales with 34–84 black body rings that are separated by white or yellow rings, except for the second head ring and three or four of the tail rings, which are bright red, hence the popular names of *cabeza de chocho* (referring to the red head of the seed of the legume *Erythrina rubrinervia*), *coral rabo de ají* (in English: chili pepper tail coral snake) or red-tailed coral snake [4] (Fig 1).

Timid, nocturnal and mostly subterranean, this *M. mipartitus* tends to hide under weed, fallen leaves and grasses. It is oviparous and its venom is neurotoxic, with an estimated lethal dose 50% for mice (18–20 g body weight) of 9 μg (450 μg/kg) [5]. This primarily neurotoxic venom provokes progressive paralysis by neuromuscular blockade and, if the inoculum is sufficient, death due to respiratory arrest. The treatment is performed with specific antivenom associated with respiratory support including intensive care in special cases [6]. In this report, we describe the clinical presentation and management of two patients bitten by red-tailed coral snakes, treated at a referral hospital in southwest Colombia.

## Case presentations

Between 2005 and 2015, 97 patients were admitted at Fundación Valle del Lili, a tertiary care university hospital in Cali, Colombia, due to snake bites: 12 were bitten by nonvenomous snakes (including non-venomous mimics), 79 by *Bothrops* and *Bothriechis* snakes and six (7%) by red-tailed coral snakes (*M. mipartitus*). Snakes were identified and classified by a biologist based on the dead body that is usually brought by patients. *M. mipartitus* were classified according to phenotypic characteristics [7]. Although in the geographical area of interest there are other species of *Micrurus*, there are no records of patients bitten by them during this period. False corals are present in the area and their bites cause minor abrasions. Clinical records of these six patients afflicted by red-tailed coral snake bites were reviewed: three were asymptomatic (had no neurological signs of envenomation, possibly due to dry bites) and three suffered envenomation with neurological involvement. Herein, we describe the clinical characteristics and treatment of the two representative cases. The patients were bitten in the rural area of Jamundí, a city located 24 km away from Cali, Colombia.

### Case 1

A 46-year-old female was accidentally bitten on the outer surface of her left foot by a *M. mipartitus* snake. The length of the animal could not be measured because its body was fragmented and some parts were lost. The accident occurred in March 31, 2005 at 7:30 a.m. Thirty minutes after the bite she complained of moderate pain and progressive paresthesia in the foot and leg. After that, she developed dysarthria, bilateral palpebral ptosis, difficulty walking and decreased strength in the upper limbs. Two hours after the bite, she was admitted to our hospital.

**Fig. 2:**
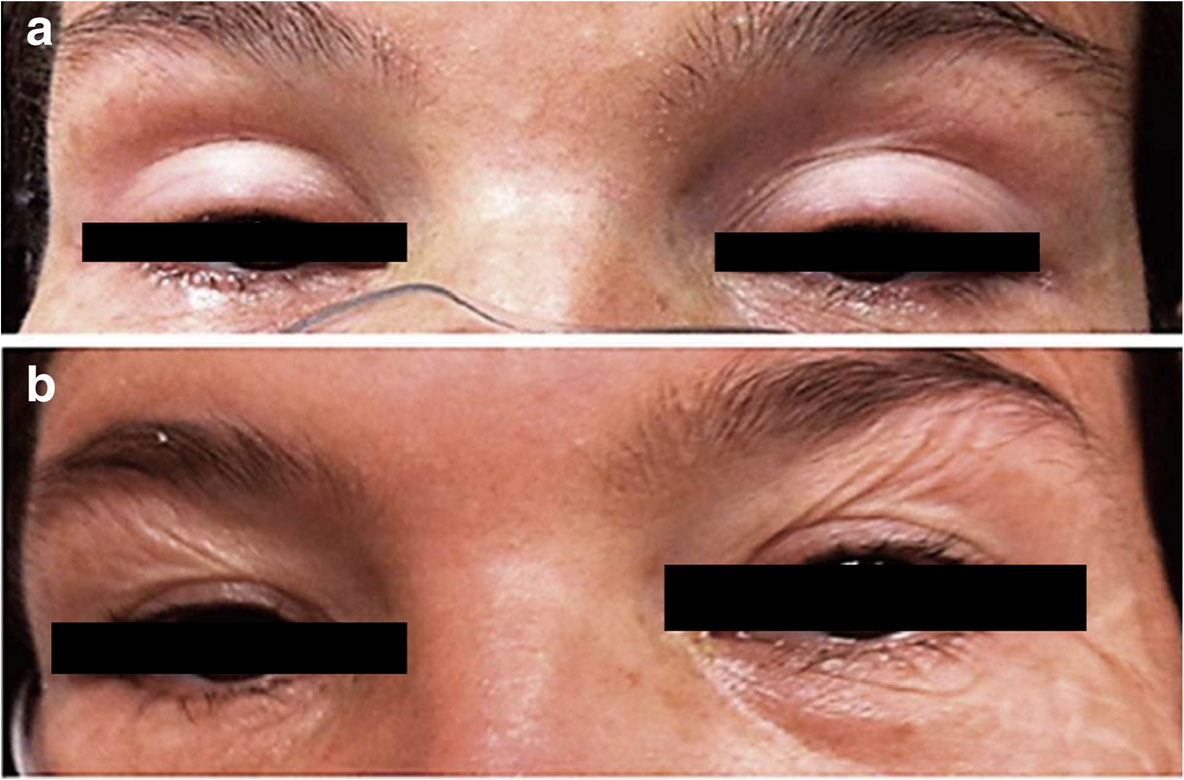
**a** Case 1, woman bitten by *M. mipartitus* with characteristic palpebral ptosis. **b** Total neurological recovery after treatment

On physical examination blood pressure was 100/72 mmHg, pulse was 84 beats/min, temperature was 36.7 °C, O_2_SAT 82% and FiO_2_ 21%. Neurological findings were severe difficulty speaking, bilateral palpebral ptosis (Fig. 2a), flaccid quadriparesis and quickly progressive respiratory depression. There was diminished chest expansion. Her skin exhibited two small puncture wounds on the outer edge of left foot, associated with mild edema, with no signs of bleeding. Laboratory exams revealed that blood count, erythrocyte sedimentation rate, C-reactive protein, coagulation, renal and hepatic function were normal. Blood gases showed PO_2_ 45 mmHg, PaCO_2_ 48 mmHg and pH 7.25. Endotracheal intubation and respiratory support were required.

Eight vials of monovalent coral antivenom (each vial containing 10 mL of equine lyophilized coral antivenom, which neutralizes at least 1 μg of snake venom, manufactured by Laboratorios Probiol SA, Colombia) were administered. No adverse reaction was observed. Six hours after antivenom therapy, the blood gases (PO_2_ 62 mmHg, PaCO_2_ 40 mmHg and pH 7.41), respiratory parameters and neurological examination were all normal, and the patient was extubated (Fig. 2b). She remained hospitalized for 24 additional hours for clinical observation, after which she was discharged asymptomatic, with a normal physical examination.

### Case 2

On October 8, 2010 at 8:30 a.m., a 22-year-old male soldier was accidentally bitten by a red-tailed coral snake of 72 cm in length at the first interdigital space of his right hand. Thirty minutes after the bite he referred mild pain and paresthesia on the right hand. Subsequently, he developed dysarthria and bilateral palpebral ptosis. Three hours after the bite he presented difficulty walking and progressive respiratory distress. Military physicians started bag-mask ventilation, and the patient was transported by helicopter to our hospital. On physical examination, blood pressure was 120/75 mmHg and pulse was 94 beats/min. Neurologic examination demonstrated bilateral palpebral ptosis and flaccid quadriparesis. Cardiopulmonary examination revealed diminished chest expansion. A puncture wound on the first interdigital area of right hand was present, with no edema or signs of bleeding. Full blood count, erythrocyte sedimentation rate, C-reactive protein, coagulation, renal and hepatic function tests were normal. Blood gases showed hypoxemia and moderate hypercapnia (PO_2_ 40 mmHg, PaCO_2_ 50 mmHg).

The patient was then intubated and respiratory support was administered. Six vials of monovalent antivenom (each vial containing 10 mL of equine lyophilized coral antivenom, which neutralizes at least 1 μg of snake venom, manufactured by Laboratorios Probiol SA, Colombia) were applied. The patient had progressive improvement of respiratory parameters, neurological examination and blood gases; therefore extubation was decided 20 h after the admission. He was discharged from hospital 24 h later.

## Discussion

Envenomation by *M. mipartitus* provokes paralysis due to the neuromuscular blockage of nicotinic acetylcholine receptors elicited by an α-neurotoxin member of the three-finger toxin superfamily [8]. A component of this venom is called mipartoxin-I, which presents the characteristic cysteine signature and amino acid sequence length of the short-chain type-I α-neurotoxins [8, 9]. Mipartoxin-I has shown a potent lethal effect on mice (intraperitoneal median lethal dose: 0.06 μg/g body weight), and causes a strong neuromuscular blockade on both avian and mouse nerve-muscle preparations, presenting a post-synaptic action through the cholinergic nicotinic receptor. *M. mipartitus* venom provokes a concentration dependent inhibition (3–10 μg/mL) of nerve-mediated twitches and significantly inhibits contractile responses to exogenous ACh (1 mM), but not to KCl (40 mM), which indicates a postsynaptic mechanism of action [9].

Since mipartoxin-I is the most abundant (28%) protein in *M. mipartitus* venom, it has been postulated that it should play a major role in its toxicity [10]. Anticholinesterase agents such as neostigmine have proven to reverse the effects of *M. frontalis* venom on animal model and may be useful in the treatment of some patients [11]. These agents were not employed in the treatment of *M. mipartitus* envenomation of the present report.

The patients of this report exhibited a predictable clinical pattern. Typically, bites affected hands or feet, places reachable by these species. Bite sites presented small skin disruptions, and mild to moderate pain and mild edema. In a first phase of the envenomation, minutes after bites, patients complain of local pain and paresthesia. Next, depending on the inoculum, systemic neurological envenomation appears. In severe cases, neurological manifestations are present within 30 min after the bite. In the second neurological phase, symptoms may delay one or two hours. Patients usually present progressive bilateral ptosis and difficulty speaking and later progressive loss of muscle strength in extremities and difficulty walking. They may develop other manifestations including salivation and drowsiness. Afterwards, there is respiratory paralysis. Neurointoxication, then, first affects cranial nerves synapses, and only later nerves controlling respiratory muscles. Flaccid quadriparesis usually occur. A third phase (severe flaccid quadriplegia) comes when the patient has been kept alive by assisted ventilation. Without treatment, *M. mipartitus* envenomation can be lethal. Fortunately, specific antivenom reverses the effects of envenomation. If the patient receives only mechanic ventilation, reversion of paralysis may take several weeks [12]. Table 1 shows symptoms and signs of our two patients in relation to time after bites.

**Table 1:** Clinical manifestations of envenoming by *M. mipartitus* in two patients

Gender, age, bite site	Phase 1 – Initial manifestations	Phase 2 – Neurological symptoms and signs before respiratory arrest – Treatment	Phase 3 – Neurological symptoms and signs after respiratory arrest with patient with respiratory assistance
Case 1, female, 46 y.o., left foot	Onset: 30 min. after the bite, moderate local pain and paresthesia	Onset: One hour after the bite, she shows dysarthria and bilateral palpebral ptosis Two hours after the bite: difficulty walking and decreased strength in the upper limbs Three hours after the bite: severe difficulty speaking and progressive respiratory depression – respiratory support and antivenom	Progressive recovery. Extubation: six hours after the antivenom application
Case 2 male, 22 y.o., right hand	Onset: 30 min. after the bite, local pain and paresthesia	Onset: Two hours after the bite, he developed dysarthria and bilateral palpebral ptosis Three hours after the bite: he presented difficulty walking and progressive respiratory distress – respiratory support and antivenom	Progressive recovery. Extubation: 20 h after the antivenom application

*y.o.* years old; *min.* minutes

Cases of envenomation by *M. mipartitus* from Colombia had been previously reported. In 1987, Angel-Mejia R. [13] described three cases from the northeastern region of the country. The first victim was a 5-year-old girl with mild envenomation that recovered without respiratory support. The second was a woman of 27 years old who had a severe envenomation and was admitted to the hospital 15 h after the bite with severe respiratory distress. She was treated with mechanical ventilation and nine vials of Costa Rica antivenom, and presented progressive recovery. The third victim was 50-year-old man treated with mechanical ventilation who did not receive antivenom and recovered after 14 days. An extension of the latter historic case occurred in the city of Manizales in 1968 and was recently reported [13]. Otero-Patiño et al. [14] and Badillo et al. [15] reported two additional cases from northwestern and northeastern Colombia, respectively. Herein, we described two new cases of envenomed patients by red-tailed coral snake, illustrating its clinical presentation and outcome with antivenom treatment.
